# Reliability of Smartphone Applications for the Quantification of Oxygen Saturation

**DOI:** 10.7759/cureus.19417

**Published:** 2021-11-09

**Authors:** Almas F Khattak, Susan S Kakakhel, Noman K Wazir, Madiha Khattak, Tania Khattak, Faryal Akbar

**Affiliations:** 1 Community Medicine and Research, Northwest School of Medicine, Peshawar, PAK; 2 Physiology, Northwest School of Medicine, Peshawar, PAK; 3 Psychiatry, Northwest General Hospital and Research Center, Peshawar, PAK; 4 Physiology, Khyber Medical College, Peshawar, PAK; 5 Pathology, Frontier Medical College, Abbottabad, PAK

**Keywords:** smartphone health applications, reliability, oxygen saturation, technology, healthcare, bland-altman plot, oximetry, covid-19

## Abstract

Background

Smartphone technology is rapidly evolving and advancing, with many of them offering health applications being used for oximetry purposes, including the Samsung Health/S Health application. Measuring oxygen saturation is one of the important indications to monitor patients with COVID-19, as well as other health conditions. These applications can be used for measuring oxygen saturation to provide a convenient solution for clinical decisions.

Methods

Oxygen saturation measurements were collected using the Samsung Health application for Samsung Galaxy smartphone with a sensor and camera flash and a low-cost portable digital display (liquid crystal display (LCD)) finger pulse oximeter. Intra-session reliability was established to determine the consistency between the measures. Intra-class correlation coefficients (ICCs) were calculated with 95% confidence intervals (CIs) reported for both methods. The Bland-Altman plot was used to compare the level of agreement between the two measurement methods.

Results

There was a statistically significant average difference between pulse oximeter and Samsung Health application measurements (t_125_ = 4.407, p < 0.001), and on average, pulse oximeter measurement was 0.510 points higher than Samsung Health application measurement (95% CI = 0.281-0.740). The pulse oximeter and Samsung Health application scores were moderately correlated (r = 0.462). The results of the intra-session reliability test produced an acceptable ICC value of 0.557, indicating moderate reliability and consistent results for the measurement of oxygen saturation with both methods. The Bland-Altman plot showed a consistently equal distribution of data points scattered above and below zero.

Conclusion

Smartphone health applications can be used with moderate reliability to measure oxygen saturation.

## Introduction

The COVID crisis is requiring us to manage patients with as little in-person contact as possible. The assessment of a patient with respiratory problems usually includes the measurement of blood oxygen saturation (SpO_2_) using a validated pulse oximeter [1]. This is particularly important in unwell patients with COVID-19 since hypoxia is a serious warning sign for severe pneumonia [2]. While a standard pulse oximeter placed on the patient’s finger is used in in-person assessment, few patients have such a technology in their homes. Various technology companies have developed smartphone applications that are marketed as accurate in measuring oxygen saturation [3]. Measuring oxygen saturation is one of the critical indications to monitor patients with COVID-19 to prevent them from going into hypoxia as a decrease in oxygen saturation could prove fatal especially in those with respiratory symptoms. In such conditions, smartphone technology can come in handy to provide convenient solutions in patient care [4-6]. Many health-related dynamics have drastically changed around the world due to COVID-19, and the shortness of medical equipment is no exception. At the peak of the pandemic, the world witnessed an extreme and desperate shortage of something as common as a finger pulse oximeter. Hence, we came up with the idea of assessing a smartphone application as oximeters to provide an alternate convenient method of oxygen saturation measurement to make up for the shortage of such devices in desperate emergency situations. Moreover, we also anticipated this to be a readily available method of measuring oxygen saturation at home settings to monitor those patients who do not need hospitalization. Our research question was, "Are smartphone applications as accurate and reliable as portable oximeters in measuring oxygen saturation in healthy volunteers at rest?" To answer this question, we conducted a study with the prime objective of comparing a smartphone application in measuring oxygen saturation with a portable finger pulse oximeter.

## Materials and methods

Adult healthy individuals of both genders, aged between 18 and 75 years, without any disease state, were recruited in this study after informed written consent was obtained. A total of 126 healthy volunteers, conveniently sampled, working in Northwest School of Medicine, Peshawar, Pakistan, and its affiliated hospitals participated in this cross-sectional study. Those individuals with any diagnosed respiratory illness, active disease state, and any scars, cuts, and any other abnormality in the digital area of the index finger were excluded to participate in the study. Ethical approval for this study was obtained from the ethics committee of Northwest General Hospital and Research Center, Peshawar, Pakistan (NWGH/Res/ethics/49). The total study duration was about six months.

Demographic and physical data of patients were collected, including age, gender, height, weight, and body mass index (BMI). The data for oxygen saturation was collected using a smartphone (Samsung Health/S Health) application for Samsung Galaxy phone, which uses the sensor and the camera flash to measure oxygen saturation when the index finger is held to the camera for 10-30 seconds, and a low-cost portable digital display (liquid crystal display (LCD)) finger pulse oximeter. We anticipated this unique feature of Samsung to be of great value especially in the wake of COVID-19 where minimal physical contact is recommended with patients. Thus, we embarked upon determining its reliability to provide an alternative and convenient method of quantifying oxygen saturation in patients. Oxygen saturation was measured repeatedly thrice using both methods. To keep the measurements uniform, each participant was comfortably seated for at least 10 minutes before taking measurements with the index finger on the right hand.

Data analysis

Data were analyzed anonymously with complete privacy and confidentiality. Descriptive analysis was performed for qualitative variables using percentages. The difference in measurement between both methods was computed as a new variable as follows:

Difference of Measurement = Portable Oximeter Measurement - Samsung Health Measurement

The mean score of both methods was also calculated as a new variable. These were calculated to construct the Bland-Altman plot for comparing the agreement level between the two methods of measuring oxygen saturation. The systematic differences between the means of the two methods (Samsung Health application and portable oximeter) were analyzed using a paired t-test. Means and standard deviations (SD) and p-value (set at 0.05) were reported for both measurements.

Intra-session reliability (the immediate test-retest reliability based on the mean of three measurements) was established by recording three consecutive measurements with each method to determine the consistency between the measures. Intra-class correlation coefficients (ICCs), using a two-way mixed-effects model (because we chose our subjects randomly and measuring methods were fixed) and absolute agreement (to obtain consistent results), were calculated with the 95% confidence intervals (CIs) reported for both methods. The ICC estimates and their 95% CI were interpreted as follows: poor reliability = 95% CI of ICC value < 0.5, moderate reliability = 95% CI of ICC value between 0.5 and 0.75, and good to excellent reliability = 95% CI of ICC value between 0.75 and 0.9.

The Bland-Altman plot was used to compare the level of agreement between the two measurement methods (Samsung Health versus portable oximeter). The mean of difference (bias) was calculated, and the upper and lower limits of agreement (LOAs) were constructed against the means of both measurements to define the 95% confidence intervals of agreement as follows:

Upper LOA = (SD × 1.96) + Mean of Difference = (1.300 × 1.96) + 0.510 = 3.058

Lower LOA = Mean of Difference - (SD × 1.96) = 0.510 - (1.300 × 1.96) = -2.038

The limits of agreement were defined a priori using the one-sample t-test to obtain the mean and SD of the difference between the two measurements. On the plot, the X-axis represented the mean of the two measurements, and the Y-axis showed the difference between the two measurements. If 95% of data points were scattered all over the place, above and below zero (limit of agreement on the plot), it was assumed that both methods were equally consistent in their measurements without bias. Moreover, a linear regression analysis was performed to evaluate any proportional bias between the two measuring methods with a p-value set at 0.05. If the beta value was closer to zero, it was assumed that there was no proportional bias.

Pearson correlations (-1 = perfectly negative correlation to +1 = perfectly positive correlation) with a p-value of 0.05 of both methods were also reported. The strength of the relationship between the two methods was assumed as follows: small/weak correlation: r = 0.1-<0.3, medium/moderate correlation: r = 0.3-0.5, and large/strong correlation: r = >0.5. 

## Results

The demographic characteristics of the participants are shown in Table 1. The paired t-test was used to determine the differences between the oxygen saturation values measured by pulse oximeter and Samsung Health/S Health application. The data is presented in Table 2. The test shows that there was a statistically significant average difference between pulse oximeter and Samsung Health application measurements (t_125_ = 4.407, p < 0.001), and on average, pulse oximeter measurement was 0.510 points higher than Samsung Health application measurement (95% CI = 0.281-0.740). The pulse oximeter and Samsung Health application scores were moderately correlated (r = 0.462).

**Table 1:** Participants’ baseline demographic information (N = 126)

Characteristics	Frequency (%)	Minimum	Maximum
Gender			
Male	73 (57.9%)		
Female	53 (42.1%)		
Age (mean + SD = 37.34 + 14.754)			
<25 years	27 (21.4)	18	73
25–45 years	69 (54.8)		
>45 years	30 (23.8)		
Height in ft (mean + SD = 5.46 + 0.316)		5.00	6.00
Weight in kg (mean + SD = 69.59 + 14.73)		43	120
BMI in kg/m^2 ^(mean + SD = 24.59 + 4.540)		12.70	42.10
Underweight (<18.5)	7 (5.6%)		
Normal (18.5–24.9)	65 (52%)		
Overweight (25–29.9)	41 (32.8%)		
Obese (>30)	12 (9.6%)		

**Table 2:** Mean value + SDs for oxygen saturation measured with a pulse oximeter and Samsung Health application

Method	Mean + SD	Mean of the difference + SD	Pearson (p-value)	95% CI of the difference
Portable finger pulse oximeter	97.94 + 0.915	0.510 + 1.300	0.462 (p < 0.001)	0.281–0.740
Samsung Health application	97.43 + 1.439			

The reliability data represented by intra-class correlation coefficient (ICC) for oxygen saturation measured with a pulse oximeter and Samsung Health application are presented in Table 3. The two methods showed moderate internal consistency with a Cronbach’s alpha of 0.59. The results of the intra-session reliability test produced an acceptable ICC value of 0.557, indicating moderate reliability and consistent results for the measurement of oxygen saturation with a portable pulse oximeter and Samsung Health application. 

**Table 3:** Intraclass correlation coefficient (ICC) of oxygen saturation using a portable pulse oximeter and Samsung Health application

Method	Mean + SD	ICC (95% CI)	p-value	Cronbach’s alpha
Portable finger pulse oximeter	97.94 + 0.915	0.557 (0.347–0.696)	<0.001	0.59
Samsung Health application	97.43 + 1.439			

**Figure 1:**
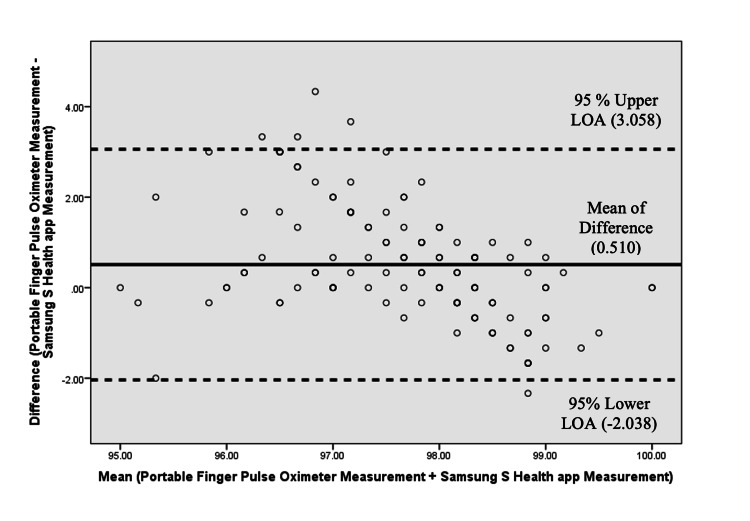
Bland–Altman plot showing the level of agreement between the two measurement methods for oxygen saturation

The Bland-Altman plot was constructed to show the level of agreement between the two measurement methods for oxygen saturation, which showed that both methods were equally consistent in their measurements of oxygen saturation as the plot showed consistently equal distribution of data points scattered above and below zero, suggesting that there is no consistent bias of one measurement method over the other (Figure 1). Furthermore, any potential proportional bias between the two methods was determined by running a regression analysis that showed a lower (beta = -0.598) unstandardized beta value for the coefficient of mean with a statistically significant result (p < 0.001), indicating proportional bias between the two methods of measuring oxygen saturation.

## Discussion

This study was conducted to determine the reliability and consistency of smartphone applications (Samsung Health application) compared with a portable finger pulse oximeter in measuring oxygen saturation in healthy adult individuals. The purpose behind this idea was that in unpredictable situations, such as the COVID-19 pandemic, when in-person contact is not encouraged, and in other situations when it is difficult to measure oxygen saturation with a finger pulse oximeter or when they are not available due to shortage, smartphone applications can be reliably used for oxygen saturation monitoring. Smartphone applications are also an easy and convenient method of measuring oxygen saturation in home settings in situations when people are monitored at home with oxygen saturation measurements.

Our findings suggested a moderate level of reliability between a portable finger pulse oximeter and the Samsung Health application for measuring oxygen saturation in healthy individuals. The reliability data suggests moderately consistent results for measuring oxygen saturation with the Samsung Health application and the portable finger pulse oximeter. The two measurement methods also demonstrated a good level of agreement for measuring oxygen saturation and positive correlation. Hence, we can suggest that these smartphone applications for measuring oxygen saturation can produce valid results, which can be used for clinical decisions in the same manner portable finger pulse oximeters are used. Jordan et al. also found a moderate agreement for SpO_2_ measurements using iPhone application; however, they conducted the study on real patients in clinical environments in contrast with our study that was conducted on healthy volunteers [7]. In situations such as the COVID-19 pandemic where healthcare settings become supersaturated and not all patients could be admitted to hospitals, oxygen saturations of otherwise stable individuals could be accurately monitored with these technologically advanced health applications in home settings, as well as clinics and hospitals. These applications are easy to use by individuals, and the findings can be interpreted by anyone without medical knowledge and the need for a health professional. Tomlinson et al. also found reliable results with iPhone applications for oxygen saturation and other oximetry measurements in healthy children and concluded that future studies should target hypoxic child patients to assess the accuracy of these applications [8]. We also recommend conducting more studies on real patients in clinical environments, hypoxic adult patients, and exercising patients to see if the smartphone applications and portable finger pulse oximeters produce the same consistency and level of agreement for oxygen saturation measurements in different settings [8-12]. Other studies termed smart technology health applications beneficial for measuring oxygen saturation in healthy adult individuals [9,13,14]. As technology is constantly evolving and advancing, it is plausible to encourage using these smartphone applications for oximetry purposes, especially in the wake of the COVID-19 pandemic, to remotely monitor patients’ oxygen saturation to prevent hypoxia in certain circumstances to preserve capacity in hospitals for more critically sick patients. The use of smartphone technology such as the Samsung Health application and other smart technology applications, where available, should be encouraged in resource-constrained settings [15-17]. This study did have limitations. First, the sample size was small and conveniently selected; therefore, the results might not be generalizable. Second, the study was conducted on healthy volunteers; hence, the findings might not be as accurate as would have been anticipated in actual patients with disease states requiring oxygen saturation measurements and monitoring. Further research in this area, specifically on actual patients, is warranted.

## Conclusions

The Samsung Health/S Heath application can be reliably used for measuring oxygen saturation as moderately consistent results and a good level of agreement were shown for measuring oxygen saturation with the Samsung Health application and the portable finger pulse oximeter. In situations such as the COVID-19 pandemic where in-person contact is not encouraged and when it is important to monitor the oxygen saturation of patients, smartphone applications can be used to make clinical decisions based on their oximetry results.
